# Accelerated peripheral vascular aging in pseudoxanthoma elasticum – proof of concept for arterial calcification-induced cardiovascular disease

**DOI:** 10.18632/aging.101821

**Published:** 2019-02-11

**Authors:** Jonas W. Bartstra, Pim A. de Jong, Wilko Spiering

**Affiliations:** 1 Department of Radiology, University Medical Center Utrecht, Utrecht, the Netherlands; 2 Department of Vascular Medicine, University Medical Center Utrecht, Utrecht, the Netherlands

**Keywords:** pseudoxanthoma elasticum, inorganic pyrophosphate, arterial calcification, vascular aging

In 1903, the German pathologist Mönckeberg described typical concentric calcifications in the medial arterial wall as a distinct phenomenon from atherosclerotic plaques. These medial arterial calcifications (MAC) have long been considered as innocent normal aging. Current treatments for cardiovascular disease target luminal thrombosis and atherosclerosis in the intimal layer. Despite widespread preventative efforts, residual cardiovascular disease burden remains high. We hypothesize that arterial calcification, especially in the medial arterial layer [1], contributes to this residual cardiovascular risk.

**Figure 1:**
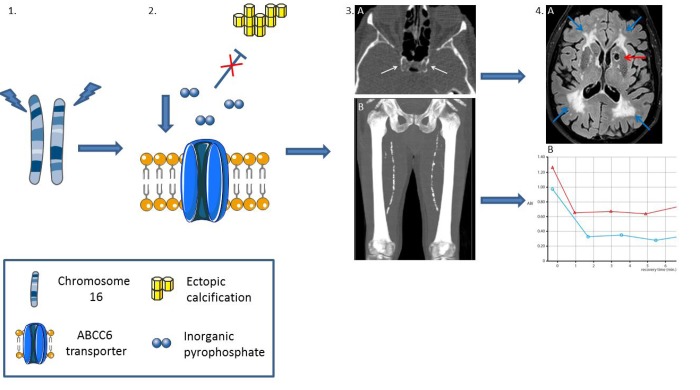
**Peripheral vascular aging in pseudoxanthoma elasticum:** mutations in the ABCC6 gene on chromosome 16 (**1**), which encodes the ABCC6 transporter, result in low levels of inorganic pyrophosphate, a strong inhibitor of ectopic calcification (**2**). The subsequent peripheral artery calcification in the carotid siphon (**3A**, white arrows) results in lacunar infarctions (4A, red arrow) and white matter lesions (**4A**, blue arrows). Calcification of the femoral arteries (**3B**) results in peripheral arterial disease (PAD). **4B** shows decreased ankle brachial index (ABI) of the left (blue line) and right (red line) leg after treadmill test, diagnostic for PAD.

Testing this hypothesis is relevant given the high prevalence of arterial calcifications in the population. Causal investigation, independent of inflammation, dyslipidaemia and thrombosis is difficult and the diagnosis of MAC is challenging as intimal and medial calcification often co-occur. In the human body a complex network of calcification promoters and inhibitors is precisely tuned to inhibit MAC [2]. Inorganic pyrophosphate (PPi) is one of the most potent calcification inhibitors in humans. It binds to hydroxyapatite crystals, thereby inhibiting further growth of the calcifications [1,2]. The consequences of disrupted PPi homeostasis are shown in the monogenetic disorders generalized arterial calcification of infancy (GACI, OMIM #208000), arterial calcification due to a deficiency in CD73 (ACDC, OMIM #211800) and pseudoxanthoma elasticum (PXE, OMIM #264800) [1]. These metabolic disorders can provide a causal disease model for MAC in the broader population. In GACI, the complete lack of PPi results in severe arterial calcification already at birth. Most affected patients die within the first 6 months of life. Patients with ACDC have severe calcification of the arteries and the joints of the hand and feet. In PXE, mutations in the *ABCC6* gene result in low circulating PPi levels [1]. This causes calcification of elastin fibers in the skin, the Bruch’s membrane of the retina and the medial arterial wall. These patients suffer from accelerated aging which results in severe visual impairment, peripheral arterial disease, gastric bleeding, ischemic stroke and cerebral white matter lesions. The arterial phenotype in PXE involves predominantly calcification of the internal elastic lamina of the intracranial arteries and the arteries of the arms and legs (Figure 1) [3].

The old bisphosphonate etidronate is a molecular homologue of PPi and has the potential to interfere in the calcification process. The drug is already indicated in GACI with a marked improvement in survival in these children and it is being tested for ACDC. We recently showed in a randomized, double blind, placebo controlled trial that one-year etidronate halts progressive calcification in peripheral arteries in PXE [4]. A larger study with longer follow-up can now proof the direct causal link between the calcification process and disease outcome in PXE to confirm our hypothesis. In addition to etidronate there is a growing number of possible compounds that can influence arterial calcification [5].

How relevant can this be for patients with diabetes, chronic kidney disease and for aging in the general population? It is clear that the residual burden and health care costs for cardiovascular disease are huge. MAC contributes to arterial stiffening which results in hypertension and heart failure, but also to pulse pressure-related damage in susceptible high flow end-organs like the kidney and the brain. Indeed increased arterial stiffness is associated with worsening of chronic kidney disease and microvascular brain damage and might therefore contribute to the development and progression of cognitive decline [6]. In the general population, MAC is shown to be the predominant type of calcification in leg arteries and probably also in the intracranial carotid artery. In the femoral and crural arteries of leg amputees, 71% of the arteries contained MAC whereas in only 31% calcified atherosclerotic lesions were seen [7]. These calcifications are the strongest predictor of major cardiovascular events such as stroke and leg amputation and also linked to dementia, heart failure and kidney failure. Probably, these ectopic calcifications have evolved as a defence mechanism against resistant infections and, in a pre-antibiotic era with a much shorter life expectancy, have aided survival and population growth. In our era, preventing and removing MAC maybe essential for healthy vascular aging, prevention of chronic cardiovascular events and multi-organ failure and might contribute to further decrease of residual cardiovascular risk.

In conclusion, there is growing evidence that MAC is a pathological and potentially treatable disease that leads to organ damage independent of atherosclerosis, inflammation and thrombosis. This is not only relevant for patients with GACI, PXE and ACDC, but also for the high residual cardiovascular risk in diabetes and kidney patients and the aging population.
